# A Chiropracticness Test

**DOI:** 10.1186/1746-1340-13-24

**Published:** 2005-11-24

**Authors:** Keith H Charlton

**Affiliations:** 1 School of Medicine, Mayne Medical School, University of Queensland, Herston, Queensland 4006, Australia

## Abstract

**Background:**

There is little homogeneity of opinion in the chiropractic profession about its essence and identity. Matters compromising the establishment of a coherent identity include the issue of vertebral subluxation, philosophy, mercantilism, poverty of qualifications in some chiropractic college faculty, and lack of intellectual productivity in some chiropractic college faculty.

**Discussion:**

The Chiropractic profession has mislabeled rhetoric, supposition and cant as philosophy, whilst showing sparse evidence for the existence of more than a few chiropractors writing in philosophy as a discipline. There is no evidence for "Chiropractic Philosophy".

I propose, however, that a better use of the discipline of philosophy can be of great use to the Chiropractic profession. Various thinkers throughout the ages have written about deduction, induction and falsificationism as methods to discover more reliably the nature of things in the world about us. Each method has strengths and frailties, but some of the latter are insurmountable for our purposes.

**Summary:**

Using a contrivance of that method which seems most suited, *sui generis*, for the purpose, I propose a Chiropracticness Test as a tool to assist the search for essence and identity in Chiropractic.

## Background

More than 100 years after its foundation, the chiropractic profession still ruminates about its eternal internal debate on "what is chiropractic?"[1]. It seems to be a profession almost uniquely divided by mostly common purposes, and even others agree significant challenges lie ahead. [2]

Amongst the most important issues denying a thematically coherent identity to portray to ourselves, to our patients, to our students, to the academy and to the world at large are:

• The issue of vertebral subluxation, an interesting hypothesis for which there is almost no evidence, yet which much of the profession, including, *mirabile dictu*, its institutions and leaders, treat as reality (and some as catechismal chant); [3]

• Whilst there is real philosophical discourse in chiropractic publications [4-8] none of it yet is by those who teach it at chiropractic colleges. The obfuscation of the issue of philosophy as if there is an entity called "Chiropractic Philosophy", when there probably is not, is a major impediment to clarity of thinking in chiropractic; [9,10]

• The rise of mercantilism, for some, placing the patients' needs second to the commercial interests of the chiropractor; [11]

• The lack of disciplinary qualifications in, say, philosophy, for chiropractic college faculty who teach in subjects of that name;

• And the failure of so many chiropractic college faculty to publish suitably in the fields in which they teach, or at all. [12]

The foregoing, and more, denies the chiropractic profession the full cultural authority to be seen legitimately as the natural custodian of the milieu in which it thinks and operates. It has failed, as says Nelson et al [13], on the front of legitimacy. Our existing institutions "have not expressed a model of chiropractic that empowers the granting of cultural authority, sustained economic viability, and scientific integrity." Can the much abused "philosophy" as a discipline help us think more usefully about these shortcomings?

## Discussion

Those immersed in the discipline of Philosophy as a vocation recognise several of its properties as characteristic: It deals with the clarification of important concepts or ideas and with clearer usage of key terms. It proceeds not by declaratory exposition, or by experimentation (there are no philosophy laboratories), but by rational endeavour, reasoning and argument. No matter how important an issue, or how wide its scope, a declaratory statement is not capable of being called philosophical unless it is defended or attacked by reasoning, not by recourse to authority, intuition or faith. Philosophy, indeed, is process.

Could Philosophy, real Philosophy, Philosophy as process, as a tool, help us here? Early last century, the great Cambridge philosopher Ludwig Wittgenstein called Philosophy a battle against the bewitchment of our intelligence by means of language [14], but I doubt he had the chiropractic profession in mind at the time.

Since this is mostly ultimately about the nature of the relationship between philosophy and science, it may help to think about how the scientific enterprise works. It seems likely that there re only two methods of rational justification: logic (deduction), and observation and experience (induction). Even these have problems. Deductive reasoning can provide no knowledge about the world around us. It offers but tautologies, or the implications of a given set of definitions and premises of, say, Euclidean Geometry. Even so, there is still no way to determine that the implications of any set of definitions and premises correspond to the complexity of observable reality.

The inductivist view is that one makes a suitably large number of observations and then distils a scientifically useful statement from the exercise. We say that if we collect large numbers of data under a wide variety of circumstances, we may endow our conclusions with greater confidence. Inductivism, the means by which most human biology research proceeds, has not escaped unscathed however. Herewith the thought processes of Bertrand Russell's inductivist turkey. It was, by all accounts, a very scientific bird, recording meticulously that it was fed every day at 9 am. It did not vary, whether the weather was bad or good, or by season. Eventually, very early one morning, the turkey felt able to claim "scientifically" "Because I have observed over a long time and under a wide variety of conditions, I now claim I will be fed every day at 9 am." Except it was Christmas Day, and at 9 am, the farmer killed the bird and the family ate it for lunch. No number of observations can exclude the possibility that a contrary future observation may render it invalid.

Because of this problem of induction exemplified by the turkey, some have felt that science needs a better way to proceed. Professor Sir Karl Popper, billed by his effusive biographer McGee as the greatest epistemologist since Aristotle, contrived falsificationism in response.

Popper made many contributions to understanding the nature of science, amongst them, the notion that no theory is ultimately provable. Perhaps his most significant contribution to the philosophy of science was his characterization of the scientific method. In *The Logic of Scientific Discovery *(1934; trans. 1959) [15], he criticized the prevailing view that science is fundamentally inductive in nature. Because he felt that reason operates primarily negatively, by criticism and refutation, he proposed a criterion of testability, or falsifiability, for scientific validity. Popper emphasized that a characteristic of a "scientific" theory is that it is vulnerable to refutation by observable events. If a hypothesis survives efforts to falsify it, it may be tentatively accepted.

Popper suggested we make precisely phrased risky conjectures about the world at large and make vigorous attempts to refute, or falsify, them. We can, he said, rely more on something we know to be false more than something we think is true because the latter may only have the status of something not yet proven wrong. If we have shown something to be false, we have learned something more useful. Truth lies somewhere in what is left. Thus, we aim for an asymptote of discovery, a verisimilitude.

Could, therefore, philosophy as process help us think about ourselves? What if we sought to phrase our claims for Chiropracticness and non-Chiropracticness in falsificationist-like prose as conjectures inviting refutation, and thus provide the meat and means for thinking about what is and is not Chiropractic? That we offer a pair of tests (The Chiropracticness Test) for what is and what is not Chiropractic at this time and in this place (it would vary).

*When we wish to know whether any proposition is true, either of chiropractic or to our purpose as a profession, we must learn whether by conceivable variation of circumstances we can cause it to break down, either by its exclusion of what we think an essence of chiropractic, or its inclusion of what we are resolved to reject as inconsistent with that essence*.

## Summary

Such a test pair would be a new approach to philosophy in chiropractic, where rumination and rhetoric have held place so far, for we might now choose to use philosophy as a tool, better to understand ourselves. The Chiropracticness Test, then, uses falsificationist means to arrive at consensus, but in ways beyond the usual method of otherwise unstructured argument for or against a matter which catches our attention. Naturally, as with any science, future knowledge cannot be known now, or it would be present knowledge, not future, and future thinkers would need to apply the Test anew as times change, and the conclusions would certainly change in the light of new observations.

Our century-long failure to address philosophy in ways familiar to those who practice philosophy as a vocation is more an issue of abstinence than impotence: we can certainly do it.

## Competing interests

The author(s) declare that they have no competing interests.
